# Integration of LaMnO_3+δ_ films on platinized silicon substrates for resistive switching applications by PI-MOCVD

**DOI:** 10.3762/bjnano.10.38

**Published:** 2019-02-07

**Authors:** Raquel Rodriguez-Lamas, Dolors Pla, Odette Chaix-Pluchery, Benjamin Meunier, Fabrice Wilhelm, Andrei Rogalev, Laetitia Rapenne, Xavier Mescot, Quentin Rafhay, Hervé Roussel, Michel Boudard, Carmen Jiménez, Mónica Burriel

**Affiliations:** 1 Univ. Grenoble Alpes, CNRS, Grenoble INP (Institute of Engineering Univ. Grenoble Alpes), LMGP, F-38000 Grenoble, France; 2 European Synchrotron Radiation Facility (ESRF), F-38054 Grenoble, France; 3 Univ. Grenoble Alpes, CNRS, IMEP-LAHC, F-38000 Grenoble, France

**Keywords:** manganite, metal organic chemical vapour deposition (MOCVD), resistive switching, thin film, valence-change memory

## Abstract

The next generation of electronic devices requires faster operation velocity, higher storage capacity and reduction of the power consumption. In this context, resistive switching memory chips emerge as promising candidates for developing new non-volatile memory modules. Manganites have received increasing interest as memristive material as they exhibit a remarkable switching response. Nevertheless, their integration in CMOS-compatible substrates, such as silicon wafers, requires further effort. Here the integration of LaMnO_3+δ_ as memristive material in a metal–insulator–metal structure is presented using a silicon-based substrate and the pulsed injection metal organic chemical vapour deposition technique. We have developed three different growth strategies with which we are able to tune the oxygen content and Mn oxidation state moving from an orthorhombic to a rhombohedral structure for the active LaMnO_3+δ_ material. Furthermore, a good resistive switching response has been obtained for LaMnO_3+δ_-based devices fabricated using optimized growth strategies.

## Introduction

Resistive switching (RS) denotes the phenomena occurring in capacitor-like heterostructures (metal–insulator/semiconductor–metal, MIM), namely memristors, when a non-volatile change of resistance is produced under the effect of an applied current or electric field [1]. As these resistance changes are reversible, RS is suitable for redox-based resistive switching random access memory (Re-RAM) applications, where different resistance values can be written, read and erased by applying the appropriate voltages. Typically two different states are characterized per device, i.e., a high-resistance state (HRS) and a low-resistance state (LRS).

As promising candidates for this application, manganites present large programming windows [2–3], i.e., a high ratio between HRS and LRS, as well as the possibility of multi-level resistance states [4], a clear advantage towards increasing storage density. The manganite of our choice is LaMnO_3+δ_ (LMO), as it is able to accommodate a wide range of cation and oxygen stoichiometry (La_1−_*_y_*MnO_3−(3_*_y_*_/2)+δ_ and LaMn_1−_*_z_*O_3−(3_*_z_*_/2)+δ_), which leads to changes in its electrical properties [5]. Here it is important to notice that for a La/Mn ratio of 1 LaMnO_3+δ_ corresponds to the simplified formula for cation deficient La_1−ε_Mn_1−ε_O_3,_ where ε = δ/(3 + δ). The apparent oxygen excess in LMO films is expected to be compensated by a mixed valence state of the manganese cation (Mn^3+^/Mn^4+^).

Particularly, RS in LMO has been reported to be larger for oxygen vacancy-rich films [6–7]. Depending on the oxygen content (δ), the LMO structure changes from orthorhombic to rhombohedral at high δ [8]. This transition occurs around δ = 0.09 at room temperature for a La/Mn ratio of 1:1 [5,9]. A similar transition from orthorhombic to rhombohedral with increasing oxygen content has also been reported for non-stoichiometric lanthanum manganites (La/Mn ≠ 1), for which, following Vegard’s law, a linear variation of the lattice constants was observed with *y* and *z* for the orthorhombic samples, while a monotonic decrease of the rhombohedral angle α was observed for the rhombohedral samples [10].

In order to grow engineered LMO films, we chose the pulsed injection metal organic chemical vapour deposition (PI-MOCVD) technique, as it allows for a controlled growth of the perovskite phase over large areas (at wafer level) with high film uniformity and conformal coverage using liquid precursors at room temperature and under an inert atmosphere [11–12]. Both conventional MOCVD [13–15] and PI-MOCVD [16–17] have been used for the deposition of epitaxial and polycrystalline LMO and doped LMO thin films enabling the control of the oxygen and cation stoichiometry.

Since the precursor solution is simply prepared by dissolution of metalorganic species, the stoichiometry of the film is easily tuneable by changing their concentration. Besides, the PI-MOCVD technique offers the additional benefit of injecting micro droplets by using an electric valve granting excellent control over the quantity of precursor transferred to the reaction chamber and therefore a good control of the thickness of the films. Hence, by modifying a number of controlled parameters on the process, such as pulse frequency, oxygen partial pressure and temperature, the structure of the LMO thin films can be tuned during growth.

In memristors, the electrodes play a crucial role in the RS response. For example, in electrochemical metallization memory chips, ions from the electrode (such as Cu or Ag) [18–19] migrate to the other electrode generating a filament across the memristive material. Another example is the case of valence-change memories, in which the nature of the contact varies depending on the difference between the work functions of electrode and active material, creating an ohmic contact or a Schottky barrier. Furthermore, some electrodes can be oxidized forming a new interface layer that can also act as oxygen reservoir (e.g., Ti, TiO_2_) [20–22]. The use of Pt as bottom electrode in our LMO-based devices guarantees an inert and ohmic contact, as the work function of LMO is 4.5–5.1 eV [23] and the one of Pt is 5.9–6.2 eV [24].

In order to integrate crystalline LMO memristive films in silicon-based devices, we used commercial platinized silicon as the bottom electrode/substrate heterostructure. Nevertheless, these substrates undergo dewetting when exposed to high temperatures for long times. The high temperatures required to grow perovskite thin films by techniques such as PI-MOCVD or PLD are a drawback because the continuity of the Pt bottom electrode can be lost [25]. In this work, we explore a number of different strategies to integrate LMO films on platinized silicon-based devices by PI-MOCVD, overcoming the challenge of the high temperatures required for their deposition. The growth parameters have been optimized to fabricate homogeneous and dense LMO films with different values of δ. Furthermore, we report the experimental proof of structural changes related to the growth strategy, being able to tune the LMO structure from an orthorhombic to a rhombohedral phase, as well as the changes in Mn*^n^*^+^ valence associated to this structure.

## Experimental

### LMO deposition conditions

LaMnO_3+δ_ (LMO) thin films were grown by pulsed injection metal–organic chemical vapour deposition (PI-MOCVD) in a JIPELEC reactor [26–27]. The precursor solutions were prepared using tris(2,2,6,6-tetramethyl-3,5-heptanedionato)lanthanum(III) [La(thd)_3_] and tris(2,2,6,6-tetramethyl-3,5-heptanedionato)manganese(III) [Mn(thd)_3_] commercial metal–organic precursors provided by Strem chemicals, and *m*-xylene (1,3-dimethylbenzene) solvent from Alfa Aesar. All solutions were prepared with a total metallic precursor concentration of 0.0225 M and a La/Mn precursor ratio of 2, the value of which was previously optimized to grow stoichiometric films with a La/Mn ratio close to 1.

The injection of the liquid precursors was performed using a frequency of 2.5 Hz and an opening time of 2 ms with a solution feeding rate of 0.35 mL/min. The evaporator was thermalized at 250 °C and Ar was used as carrier gas. The total pressure in the chamber was fixed at 5 Torr and oxygen gas was added directly in the reaction chamber to obtain an oxygen partial pressure of 50%. Independent heaters and several thermocouples distributed along the reactor circuit allowed for a well-controlled heat gradient from the injector to the reaction chamber, maximizing the flux of carried precursor. The deposition temperature inside the main chamber (a hot-wall quartz reactor heated by an external furnace) ranged from 500 to 750 °C. The substrates used were 1 cm × 1 cm chips cut from a Pt (150 nm)/TiO_2_ (40 nm)/SiO_2_ (500 nm)/Si (111) wafer (VinKarola Instruments).

### Structural and electrical characterization

Scanning electron microscopy (SEM) was performed in a Quanta250 environmental SEM FEG from FEI, and SEM FEG ZEISS GeminiSEM 500 to study the surface morphology and determine the LMO thickness using the cross section of the films. The cationic film composition was analyzed by semi-quantitative energy-dispersive X-ray analyses (EDX) using an Oxford Inca Energy detector coupled to the SEM. A combined study in X-ray diffraction (XRD) and Raman spectroscopy was performed to determine the crystal structure of the films and to detect the presence/absence of secondary phases. XRD was measured in grazing incidence configuration (GIXRD) in a 5-circle Rigaku Smartlab diffractometer to enhance the diffraction signal from the polycrystalline films and minimize the signal of the platinized silicon substrate. Raman spectra were collected using a Jobin Yvon/Horiba Labram spectrometer equipped with a liquid nitrogen-cooled CCD detector. Experiments were conducted in the micro-Raman mode in a backscattering geometry using a green laser (λ = 514.5 nm). The silicon spectrum at ambient temperature was always measured and used as reference to calibrate the LMO spectra. The nanostructure growth was further analyzed in cross section by transmission electron microscopy (TEM), a JEOL 2011 equipment operating at 200 kV with a 0.19 nm point-to-point resolution.

X-ray absorption near-edge spectroscopy (XANES) spectra at the Mn K-edge of LMO thin films were collected at the ESRF ID12 beamline (Grenoble, France). Measurements were taken under vacuum at 25 °C in fluorescence mode using a nearly constant 200 mA beam current. Two silicon photodiodes detectors were used to collect the total fluorescence, one in back-scattering geometry and a second diode mounted at 90° with respect to the incident beam. The relative variation in the Mn formal valence was carried out from the experimental recorded inflection point after XANES spectra normalization, and using references found in the literature for other manganite perovskites [28].

The device fabrication for electrical measurements was performed in clean-room facilities combining laser lithography (Heidelberg instruments µPG 101) to define the top electrode pads (200 µm squared pads) and metal evaporation (Plassys MEB_550_ electron gun 10 kW) to grow 100 nm thick Au layer as top metal electrode. The electrical characteristics were measured under ambient conditions within a Faraday cage with microprobe manipulators using a B1500 Agilent semiconductor parameter analyzer.

## Results and Discussion

Dense and homogeneous LaMnO_3+δ_ (LMO) thin films with variable oxygen content (δ) have been deposited by pulsed injection metal organic chemical vapour deposition (PI-MOCVD). The structural transition between orthorhombic and rhombohedral phases has been correlated with δ and the manganese oxidation state of the films. Furthermore, the tuned LMO films integrated in a silicon-based substrate showed resistive switching behavior as a proof-of-concept of the suitability of their use as ReRAM.

### Optimisation of the deposition conditions

In this section we present the optimisation of the temperature and number of pulses using a fixed pressure of 5 Torr, a gas mixture composed of Ar 50% and O_2_ 50% during the deposition step, a pulse injection frequency of 2.5 Hz, and an opening time of the valve of 2 ms. These last two parameters allowed for a good evaporation of the precursors and a constant flux during deposition.

**Figure 1:**
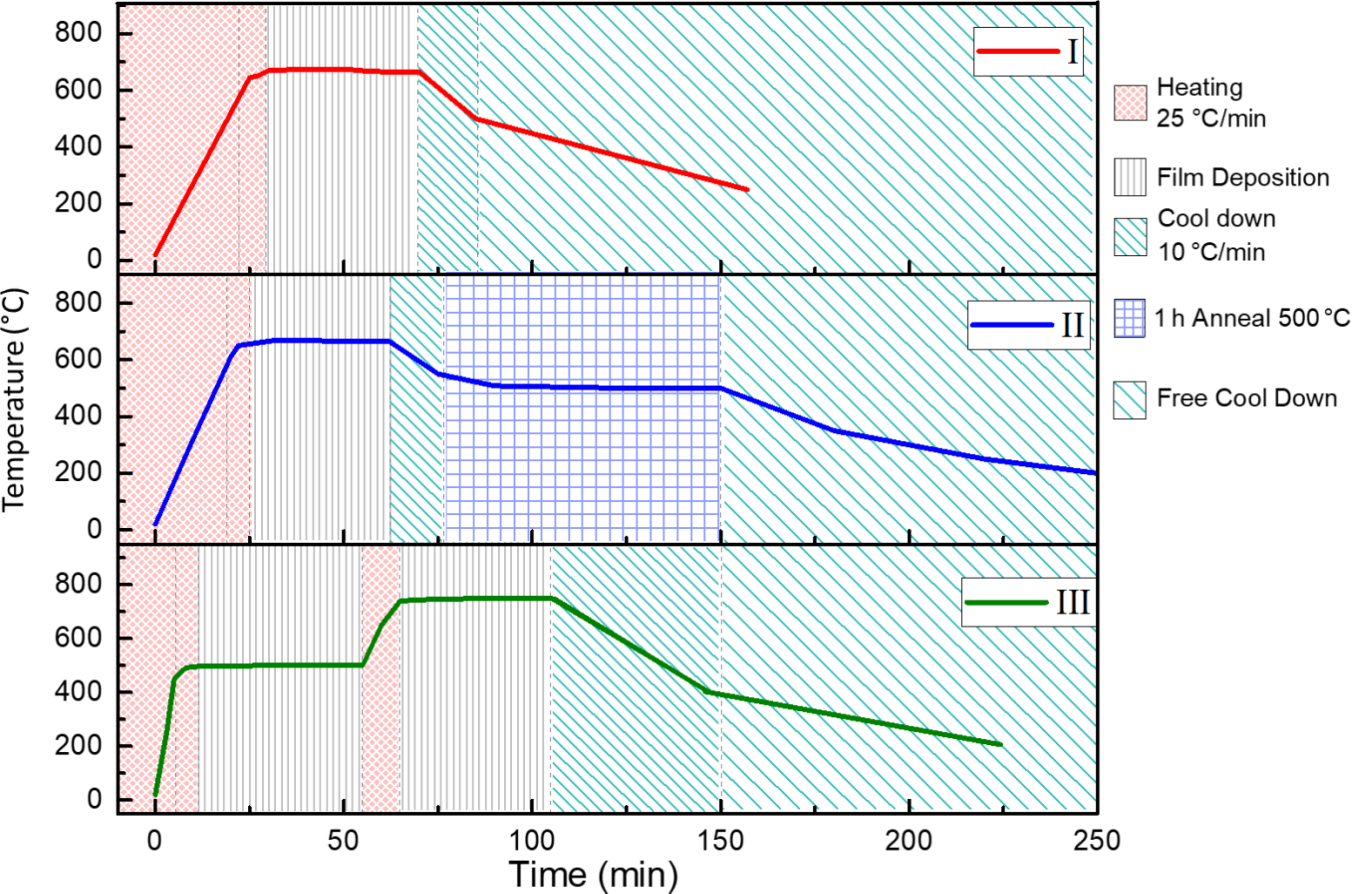
Growth diagrams of the three deposition strategies. The deposition steps correspond to temperature plateaus at 5 Torr using a gas mixture of 50% O_2_ + 50% Ar. I) single-step strategy; II) single-step strategy with an annealing treatment (i.e., anneal in oxygen) at 500 °C and 5 Torr; III) two-step strategy scheme.

Samples grown at 675 °C and ca. 1 h of deposition time (i.e., 10000 pulses) evidenced the thermal instability of the platinum substrate surface. The main issues were Pt dewetting, the formation of pinholes at the LMO film, and/or LMO film cracking due to Pt grain evolution. The surface and the cross section of the heterostructures showing cracking of the sample, holes and Pt percolating to the surface are presented in Figure S1 and Figure S2, respectively, in Supporting Information File 1. In order to avoid these problems and with the aim of decreasing the time of exposure of the bare Pt surface to high temperatures, three strategies including different heating and deposition steps were proposed and tested, as shown in Figure 1.

(I) Single-step strategy: The reactor was heated at 25 °C/min in vacuum (ca. 0.15 Torr) up to the deposition temperature. Once the temperature was reached and stabilized (ca. 10 min), the gas mixture was introduced in the chamber and the injection of precursors started and ran for a limited deposition time (controlled number of pulses). Several deposition temperatures in the range of 650–700 °C were tested.

(II) Single-step strategy with annealing: The heating and deposition procedure was the same as described in strategy I, but adjusting the deposition temperature between 660 and 680 °C. Once the injection was completed, an additional thermal treatment at 500 °C was performed. The samples were let to cool down to the post-deposition annealing temperature at a rate of 10 °C/min. At this temperature the environment was modified (i.e., annealing in oxygen) and the temperature was held for 1 h.

(III) Double-step strategy: This growth procedure was divided in two deposition steps at different temperatures. The first step consisted of depositing a stabilizing layer at an intermediate temperature (500 °C) and was followed by a second deposition step at high temperature (750 °C) to achieve the complete crystallization of the film. The heating conditions of the first step were the same as described in strategy I. Next, after a new heating ramp up to 750 °C (25 °C/min), the second deposition step took place once the temperature was reached. Cooling down began right after the injection was completed to minimize the time the sample was held at high temperature.

As common points to all strategies the mixture of gases (O_2_/Ar) was introduced in the reactor chamber when a temperature 50 °C lower than the deposition temperature was reached. Controlled cool-down conditions varied depending on the desired structure (orthorhombic or rhombohedral). The samples were cooled down to an intermediate temperature (450–400 °C) using a cooling ramp of 10 °C/min in a specific gas environment (either pure Ar or O_2_/Ar mixture in strategy I, and O_2_/Ar mixture in strategies II and III). From this intermediate temperature, the cool-down rate was free and the pressure was kept at 5 Torr using only Ar gas. The influence of the atmosphere during cooling was investigated using strategy I through comparison of the effect of using either O_2_/Ar mixture gas or pure Ar.

**Figure 2:**
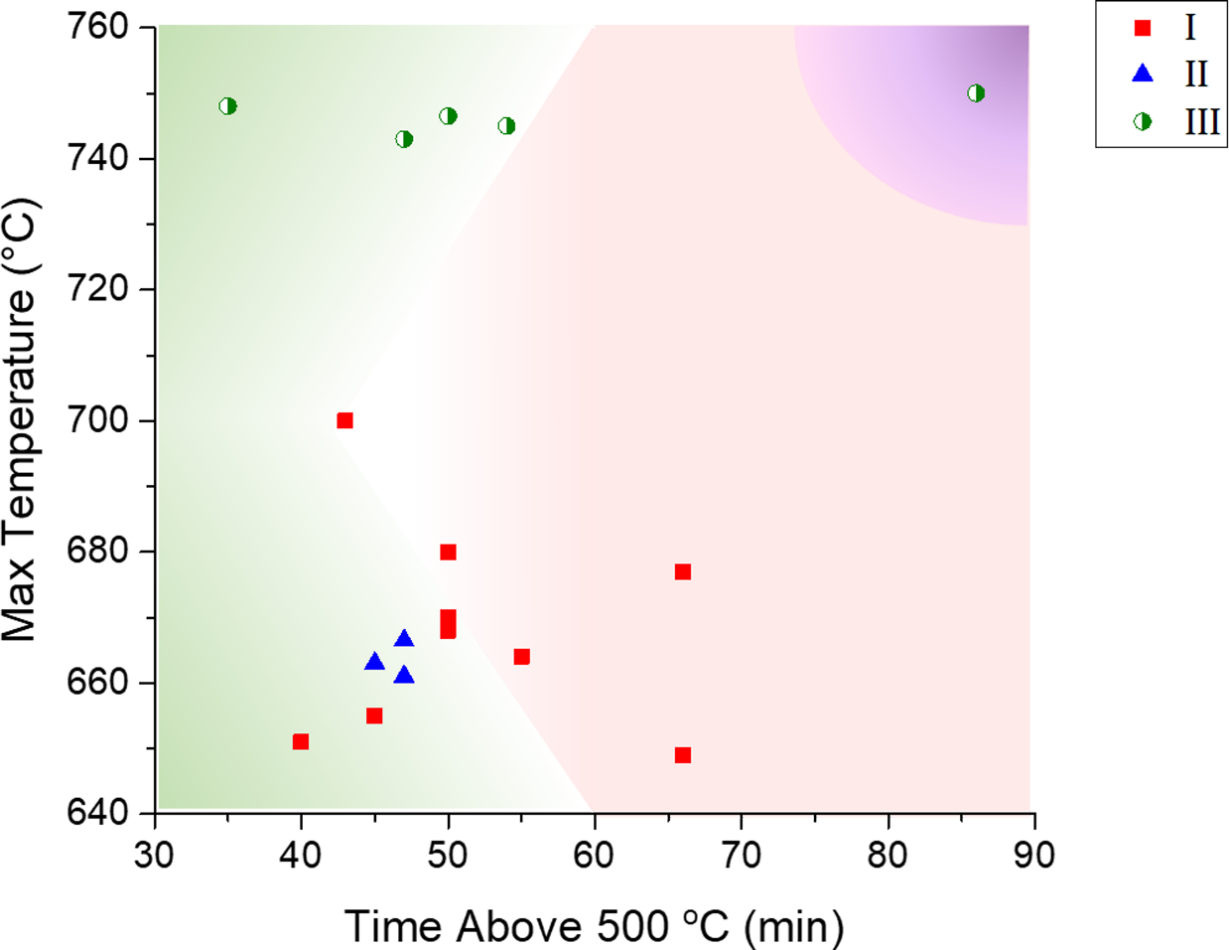
Representation of the range of appropriate deposition conditions. Maximum temperature used versus time of exposure to temperatures above 500 °C. Red squares stand for single step strategy (strategy I), blue triangles for single step strategy with in situ annealing (strategy II). Green circles stand for double step strategy (strategy III). Data in the green shadowed area correspond to successfully grown dense and homogeneous LMO films without instability issues. The red shadowed region corresponds to films with Pt instability problems. Purple area corresponds to columnar growth and opening of the grains, leading to porous films.

Figure 2 summarizes the LMO film growth as a function of the maximum temperature used and the period of time at which the samples were exposed to temperatures above 500 °C. This representation allows one to define the temperature region in which Pt remains thermomechanically stable, as well as the time limits to obtain dense and homogenous LMO films, i.e., without nanoporosities due to insufficient thickness, or the opening of grains in a flower bouquet-manner for thick films (see Figure S3 in Supporting Information File 1).

For strategy I, as a first approach samples were deposited at 500, 600 and 700 °C. At 600 °C the LMO layers showed low crystallinity whereas above 700 °C, diffusion of Pt across the film occurred, which may lead to short circuits in the final LMO-based device. The samples corresponding to this strategy are depicted as red squares in Figure 2. The points inside the shadowed green region correspond to deposition conditions for which dense and homogeneous LMO films are successfully obtained. The maximum appropriate temperature was 700 °C for times (above 500 °C) shorter than 45 min. This time limit could be increased (up to 50 min) for the deposition temperature range between 640 and 680 °C.

Strategy II was designed to obtain higher δ values in the LMO film by adding a post-deposition annealing step. LMO films were grown following strategy II (represented in Figure 2 by blue triangles) respecting the same critical limits established from strategy I. It was proved that despite the addition of a thermal annealing at 500 °C for 1 h, the selected conditions led to dense and homogeneous LMO films.

For strategy III, based on two consecutive deposition steps at 500 and 750 °C, the critical parameters were: (i) the LMO layer thickness obtained by growth at low temperature (*d*_1_) to stabilize the Pt layer, and the LMO layer thickness corresponding to the second growth at 750 °C (*d*_2_) that must fully cover the LMO film deposited first; (ii) the time at 750 °C, which must be sufficiently long to allow the bottom LMO layer to be fully crystallized but short enough to avoid Pt dewetting; (iii) the maximum thickness *d*_1_ + *d*_2_ (and the ratio between both thicknesses *d*_1_/*d*_2_) in order to obtain non-porous films (see Figure S2 in Supporting Information File 1), which is limited by the opening of grains in a flower bouquet-manner through columnar growth.

As previously explained, for strategies I and II, the time above 500 °C was limited to 45/50 min and the temperature should not exceed 700/685 °C during this time. This leads necessarily to a limitation in the LMO film thickness, which was of 60–80 nm for the fixed injection frequency of 2.5 Hz. Nevertheless, this limitation cannot be extrapolated to strategy III, for which the growth is stable for longer times, since the protective layer grown at 500 °C prevents Pt dewetting and Pt grain evolution.

The deposition time at 750 °C in strategy III was varied between 35 and 85 min. It is important to notice that strategy III allowed the growth of highly dense LMO films on Pt at 750 °C for longer deposition times, the Pt film was stable for all samples. In addition, the exposure times at 750 °C were enough to fully crystallise the LMO protective layer. From this point of view, strategy III is more robust than strategies I and II. Nevertheless, large thickness samples (green circle at 85 min above 500 °C in Figure 2) exhibit columnar grain opening that makes them inappropriate for device fabrication where flat surfaces are required. Besides, in strategy III, we have to take into consideration the effect of thicknesses *d*_1_ (500 °C) and *d*_2_ (750 °C), bearing in mind that *d*_1_ should be thick enough to operate as protective layer and that *d*_2_ should be continuous enough. Therefore, for this last strategy the minimum deposition time required above 500 °C is ca. 30 min, and the maximum time will be limited by the beginning of columnar opening, around 60 min.

In summary, the outcome of this optimisation process allowed for the determination of the optimal temperature/time range of growth for each strategy, i.e., the time limit before Pt begins dewetting, films begin to crack or nanoporosities begin to form. For films thicknesses smaller than 80 nm, strategy I and II are the most suitable, while strategy III is optimal for film thicknesses above 80 nm. In the latter case, the maximal thickness before columnar grain opening depends on *d*_1_ and *d*_2_ and will be subject of further studies.

### Structural and composition characterisation of LMO thin films

**Figure 3:**
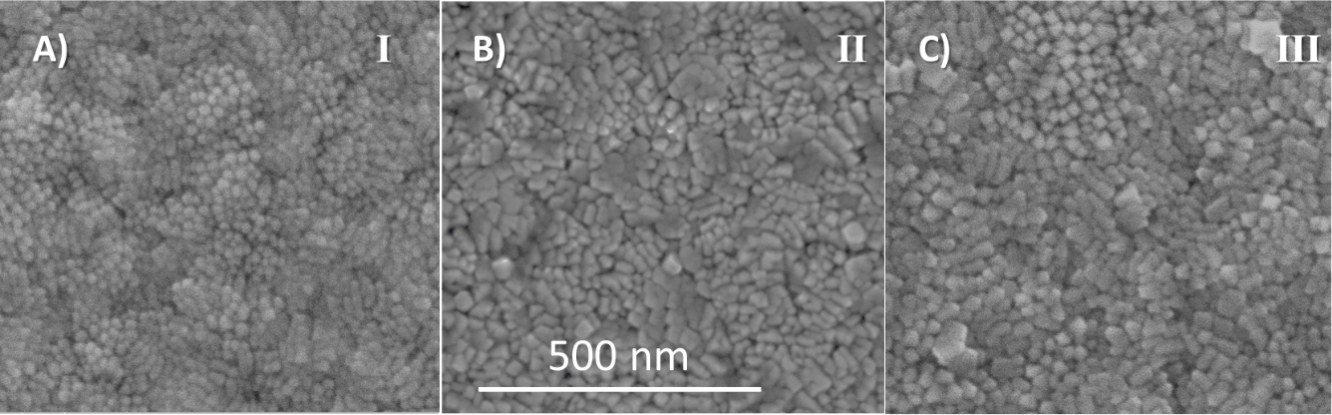
SEM surface images of the LMO films deposited by, A) strategy I, B) strategy II, and C) strategy III. The grain size increases from strategy I to III (i.e., 15, 18, 22 nm, respectively). All presented LMO thin films are of the same thickness of ca. 100 nm.

In order to properly compare the films obtained by each strategy, structural and chemical studies were carried out by electron microscopy coupled to EDX. Within the resolution limit no differences in composition were observed for the different deposition strategies. Figure 3 shows the comparison of the surface films morphology with the same thickness (80–100 nm) grown by strategy I (Figure 3A), strategy II (Figure 3B) and strategy III (Figure 3C). All films are polycrystalline and highly compact with average grain sizes of 15 ± 4 nm, 18 ± 4 nm, and 22 ± 4 nm, respectively. Moreover, the LMO films are homogeneous and do not exhibit porosity, cracking or dewetting.

**Figure 4:**
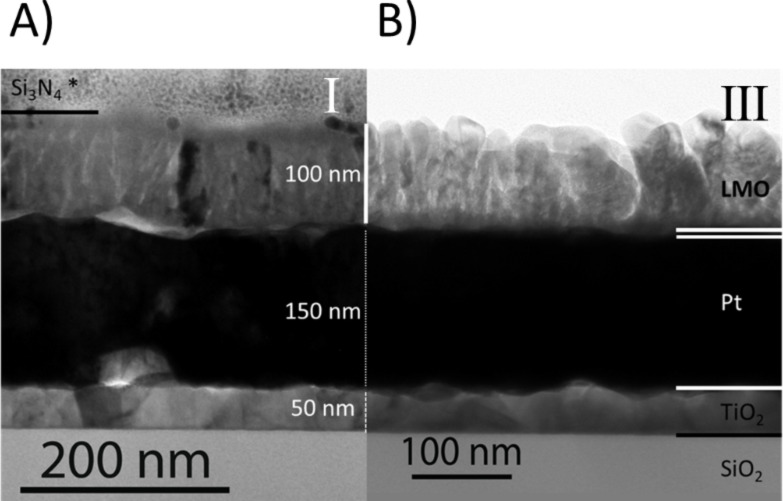
TEM cross-section images from LMO films grown by strategy I (A) and strategy III (B). A) A continuous and homogeneous film is observed. The Si_3_N_4_ coating marked with * comes from device fabrication. B) A homogeneous crystallization of LMO from bottom to top was achieved in the second deposition step.

Furthermore, TEM cross-section observation of the LMO films was performed for strategies I and III, which correspond to the extreme cases (Figure 4). The cross-section images corroborated that in both heterostructures the Pt layer is continuous and stable as required; neither dewetting nor Pt diffusion occurred at the interface. The good crystallization of both films was also verified, including the bottom part of the film for the case of strategy III (*d*_1_ thickness), which was deposited at a lower temperature. The cross section of strategy III showed more irregular columns, probably due to the difference in growth temperature between the two steps, but no flower bouquet effect is observed for this thickness of ca. 100 nm.

**Figure 5:**
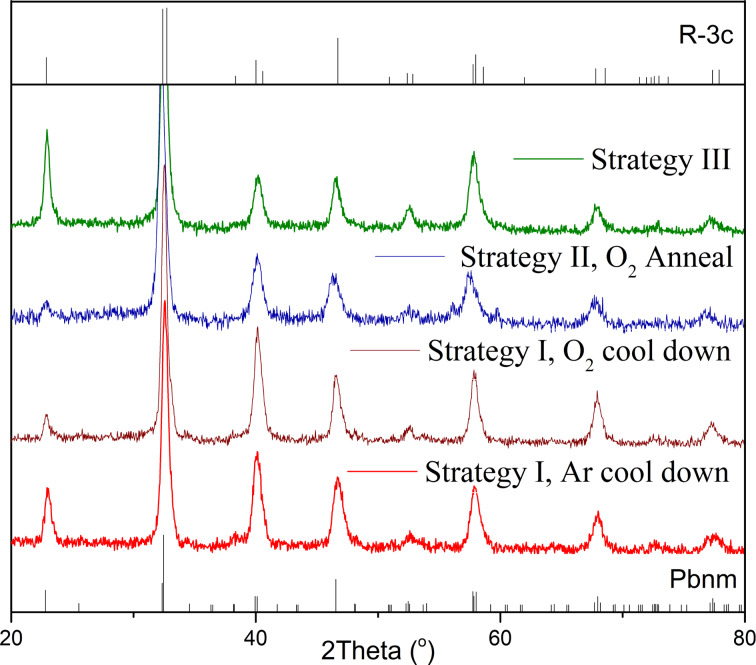
GIXRD patterns obtained for LMO thin films grown by strategy I (cooled in Ar and cooled in O_2_), strategy II (1 h post-anneal in O_2_) and strategy III. The orthorhombic (Pnma for La_0.974_Mn_0.974_O_3_) and rhombohedral (R-3c for La_0.95_Mn_0.95_O_3_) patterns have been added for comparison (ICDD: 01-087-2015 and 04-007-6350, respectively).

The first step of the phase identification for the LMO films grown by strategies I, II and III was performed by GIXRD. The XRD patterns corresponding to representative LMO films deposited by the three strategies are shown in Figure 5.

All diffraction peaks can be attributed to the polycrystalline LMO perovskite phase without any clear preferential orientation and no impurities. Therefore, pure LMO can be obtained through all three deposition strategies. Nevertheless, the orthorhombic and rhombohedral patterns are quite similar and do not allow for an easy discrimination between both structures. Because the structure is strongly linked to the oxygen content, complementary structural characterization was carried out by Raman spectroscopy to identify the LMO phase. As reported by M. N. Iliev et al. [29], Raman spectroscopy is very sensitive to small changes in the LMO structure, allowing one to discriminate between the two phases. The orthorhombic phase is characterized by sharp Raman lines at high wavenumbers: A_g_ mode (in-phase stretching/out-of-phase bending) at 493 cm^−1^ and B_2g_ mode (in-phase stretching) at 612 cm^−1^, while a weaker A_g_ mode (rotational) is observed around 257 cm^−1^. On the other hand, the Raman spectrum of the rhombohedral phase is composed of broad bands centred at ca. 497 and 617 cm^−1^, related to the Jahn–Teller distortion while weaker bands are observed at lower wavenumbers, such as the characteristic rotational mode, A_1g_, at 217 cm^−1^.

**Figure 6:**
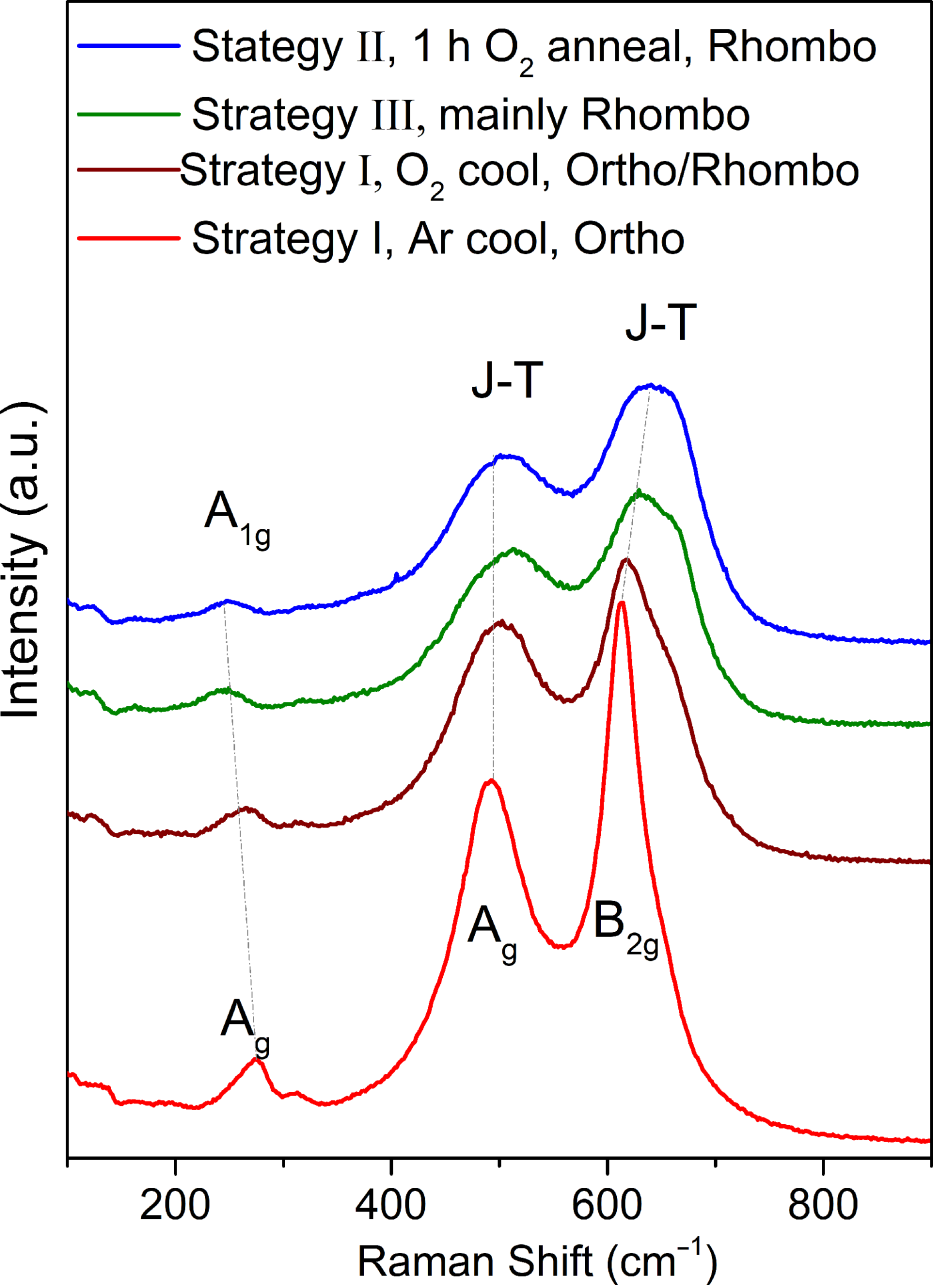
Raman spectra of LMO films obtained by the three deposition strategies under different conditions of cooling or annealing. An evolution of the structure from orthorhombic to rhombohedral phases is observed (dashed lines are guidelines for the eyes).

Figure 6 shows the evolution of the LMO Raman spectrum from an orthorhombic phase (bottom red curve) to a rhombohedral phase (top blue curve). At low wavenumbers, we can follow the evolution from the orthorhombic mode. At higher wavenumbers, a shift can be observed from the A_g_ and B_2g_ orthorhombic modes centred at 495 and 614 cm^−1^, respectively, to the broad Jahn–Teller bands characteristic of mixed-valence manganites with Mn^3+^/Mn^4+^ charge and orbital disorder [30], i.e., the first one at ca. 513 cm^−1^ and the second one split into a main band at ca. 630 cm^−1^ and a second component at ca. 660 cm^−1^ [31].

In Figure 6 we observe that pure orthorhombic and rhombohedral phases can be obtained for the two extreme deposition conditions. The LMO films obtained by strategy I show a pure orthorhombic phase when cooling down in Ar, which favours a lower oxygen incorporation in the LMO films. When the cooling is performed in O_2_, the films contain a mixture of both phases since δ increases. The annealing step incorporated in strategy II, when performed under O_2_ atmosphere, yielded a completely rhombohedral phase. Finally, the films grown using strategy III appear as mainly rhombohedral. Therefore, the Raman results give us an indication of the variation of the oxygen content in the LMO films from the structural phases observed.

**Figure 7:**
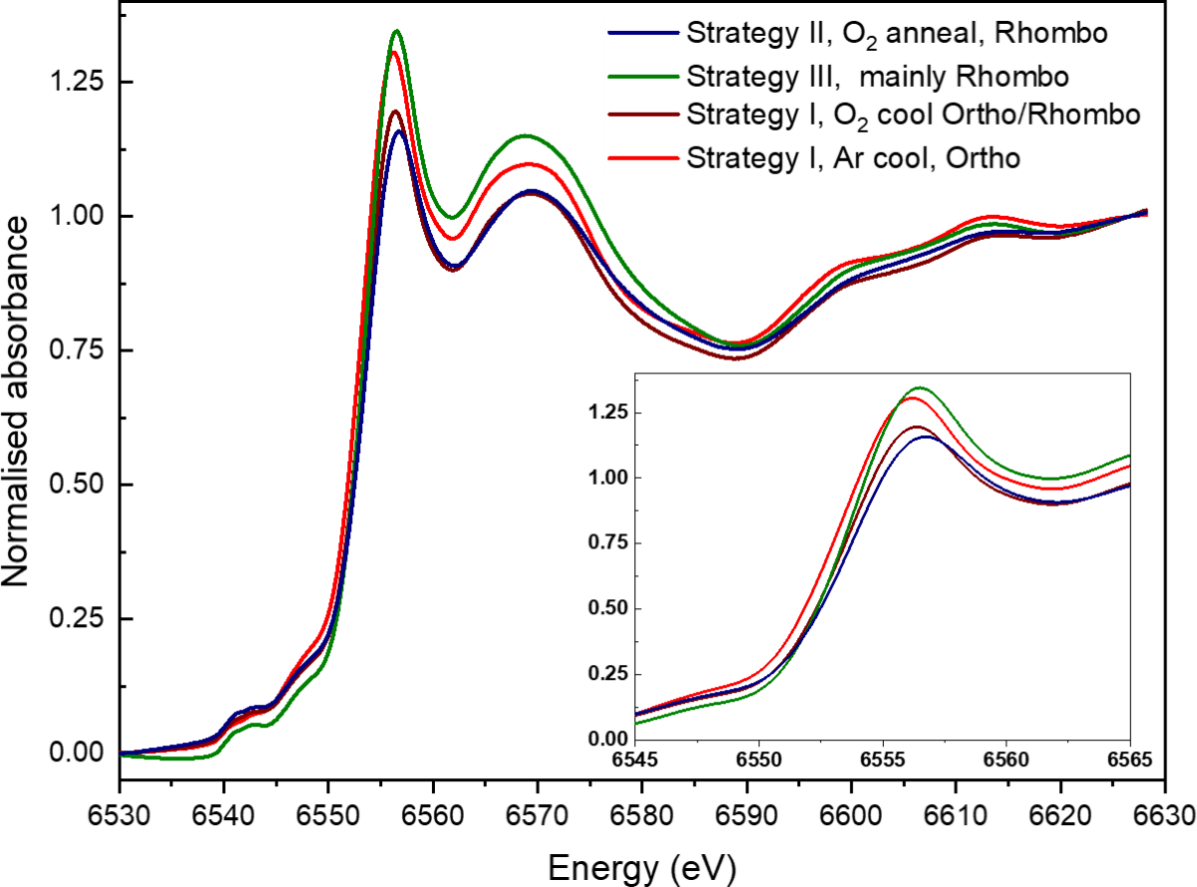
XANES absorption in Mn K-edge data of LMO films obtained by the three deposition strategies under different conditions of cooling or annealing (same samples as in Figure 6). The inset corresponds to an enlargement of the region close to the edge. An evolution of the structure from orthorhombic to rhombohedral phases is observed in good agreement with Raman spectra.

The apparent oxygen excess in LMO films is expected to be compensated by a mixed valence state of the manganese cation (Mn^3+^/Mn^4+^). The variation of the Mn oxidation state for the same series of LMO films measured by Raman was confirmed by XANES (Figure 7). The local geometry of Mn was extracted from the pre-edge feature. For all the spectra, the absence of a sharp pre-edge shape reveals an octahedral (*O_h_*) symmetry, i.e., a local symmetry of MnO_6_ units, number of coordination = 6, which is in agreement with the perovskite structure [32]. The formal valence of Mn was estimated from the Mn K-edge position using reference values reported for the LaMnO_3_ compound [28]. The Mn K-edge position was obtained from the inflection point of the absorption edges, calculated from the second derivative of the curves. The energy values obtained for the four LMO films are detailed in Table 1. As expected, the edge position increases with the Mn valence, being the lowest energy value the one of the orthorhombic LMO reference (strategy I, cooling in Ar) and the highest the one of the rhombohedral LMO reference (strategy II, annealing in O_2_). The relative variation of the Mn formal valence was estimated comparing the shift in the Mn K-edge position with the one obtained in the literature [28]. Cuartero et al. measured an energy shift of 0.4 eV for an increase from Mn^3+^ to Mn^4+^. Table 1 shows the estimated gradient in Mn formal valence among the four thin films, which is of 0.10 for the two extreme samples (rhombohedral and orthorhombic).

**Table 1:** Mn K-edge position for the four representative LaMnO_3+δ_ samples obtained by strategies I, II and III and estimated variation in the Mn formal valence taking as reference the orthorhombic sample.

**LMO deposited films**	Mn K-edge position (eV)	relative variation in Mn formal valence

strategy II (O_2_ post-anneal) rhombohedral reference	6554.26	0.10
strategy III (double step) mainly rhombohedral	6554.22	0.09
strategy I (O_2_ cooling) rhombohedral + orthorhombic	6554.00	0.04
strategy I (Ar cooling) orthorhombic reference	6553.85	0

All presented results confirm that it is possible to grow dense polycrystalline LMO films by PI-MOCVD adjusting the deposition conditions. Furthermore, we can tune the oxygen content and the resulting Mn oxidation state, leading to a structure transition from orthorhombic to rhombohedral phase. Considering the advantages and limitations of the three deposition strategies, despite the narrow temperature and time ranges required to avoid Pt instability, strategy I allows for the growth of LMO thin films up to 80 nm providing a very good control of the crystal structure. On the other hand, when rhombohedral films are suitable for the required application, the double-step strategy has proven to be more robust, allowing for the growth of thicker samples by adjusting the thicknesses *d*_1_ and *d*_2_.

**Figure 8:**
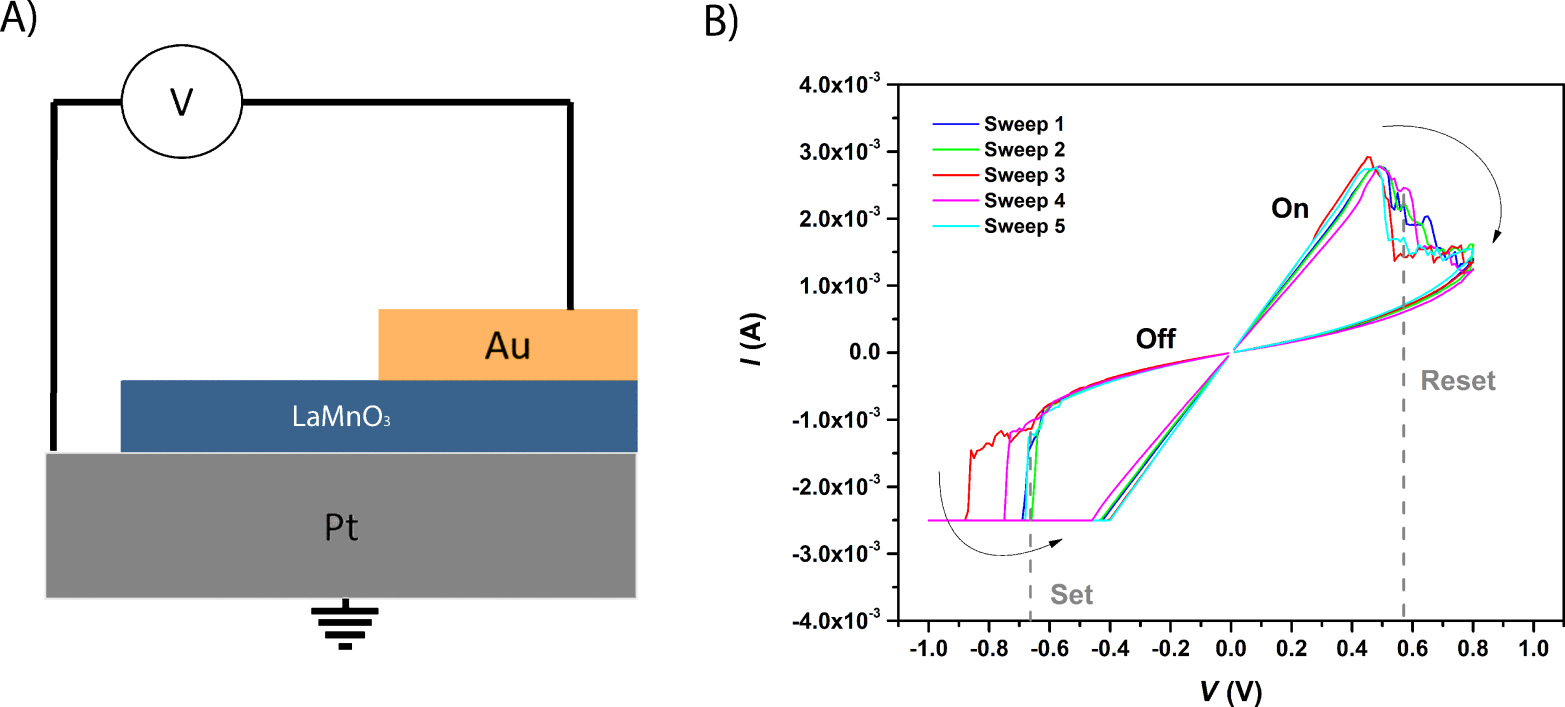
A) Cross section of the LMO-based MIM structure. B) Resistive switching cycles, *I*–*V* characteristics detailing the “set” and “reset” values.

Once the films were obtained we proceeded to the fabrication of MIM structures by evaporating squared Au electrodes of 200 × 200 µm^2^. Figure 8 presents the current–voltage characteristics of an orthorhombic LMO device. After a forming step, reproducible and reversible clockwise bipolar RS response was attained. The “set” and “reset” values were around −0.7 V and +0.6 V, respectively, which are considerably lower than those reported for epitaxial LMO (−1.5 V and +3.0 V) [6]. While the ratio of resistance of the “ON” and “OFF” states was of the same order as the one reported in the literature (over 7.3 in our case for polycrystalline LMO and between 1.8 and 18 depending on anneal conditions for epitaxial LMO [6]). Therefore, LMO films have proven their suitability as functional material for ReRAM applications.

## Conclusion

After solving the issues related to the thermomechanical instability of Pt in the substrate, LMO films have been integrated on a platinized silicon substrate by using PI-MOCVD to fabricate perovskite-based ReRAM devices. To do so, three deposition strategies have been developed. All of them offer the possibility of growing dense and homogeneous LMO films suitable for resistive switching applications. Furthermore, the oxygen content can be tailored by adjusting the deposition parameters, such as temperature, number of pulses and atmosphere used during cooling. The transition from the orthorhombic to the rhombohedral phase has been correlated with the oxygen content and the Mn oxidation state of the LMO films. Orthorhombic films, as well as films with a mixture of phases have been obtained by using strategy I. With strategy II, which includes annealing in oxygen atmosphere, it was possible to obtain a purely rhombohedral structure. The third double-step strategy has proven to be a very robust method to grow mainly rhombohedral samples of comparable and higher thicknesses than the other strategies by adjusting the thicknesses *d*_1_ and *d*_2_. Therefore, by combining growth parameters in PI-MOCVD and wisely choosing the best deposition strategy we can tune the characteristics of the LMO films for functional devices. Finally, using Au and Pt as top and bottom electrodes, respectively, the functional resistive switching properties of the optimized LMO films has been validated.

## Supporting Information

File 1 Additional figures.
