# Supplementary material for: Integration of LaMnO3+δ films on platinized silicon substrates for resistive switching applications by PI-MOCVD
Source: Beilstein J Nanotechnol. 2019 Feb 7;10:389–98. doi: 10.3762/bjnano.10.38 (PMC6369995; doi:10.3762/bjnano.10.38)
Supplement: File 1 — Additional figures. [file Beilstein_J_Nanotechnol-10-389-s001.pdf]

BEILSTEIN JOURNAL OF NANOTECHNOLOGY

Supporting Information

for

Integration of $LaMnO_{3+\delta}$ films on platinized silicon substrates for resistive switching applications by PI-MOCVD

Raquel Rodriguez-Lamas, Dolors Pla, Odette Chaix-Pluchery, Benjamin Meunier, Fabrice Wilhelm, Andrei Rogalev, Laetitia Rapenne, Xavier Mescot, Quentin Rafhay, Hervé Roussel, Michel Boudard, Carmen Jiménez and Mónica Burriel

Beilstein J. Nanotechnol. **2019,** *10,* 389–398. doi:10.3762/bjnano.10.38

Additional figures

License and Terms: This is a supporting information file under the terms of the Creative Commons Attribution License (http://creativecommons.org/licenses/by/4.0). Please note that the reuse, redistribution and reproduction in particular requires that the authors and source are credited.
The license is subject to the *Beilstein Journal of Nanotechnology* terms and conditions: (https://www.beilstein-journals.org/bjnano)

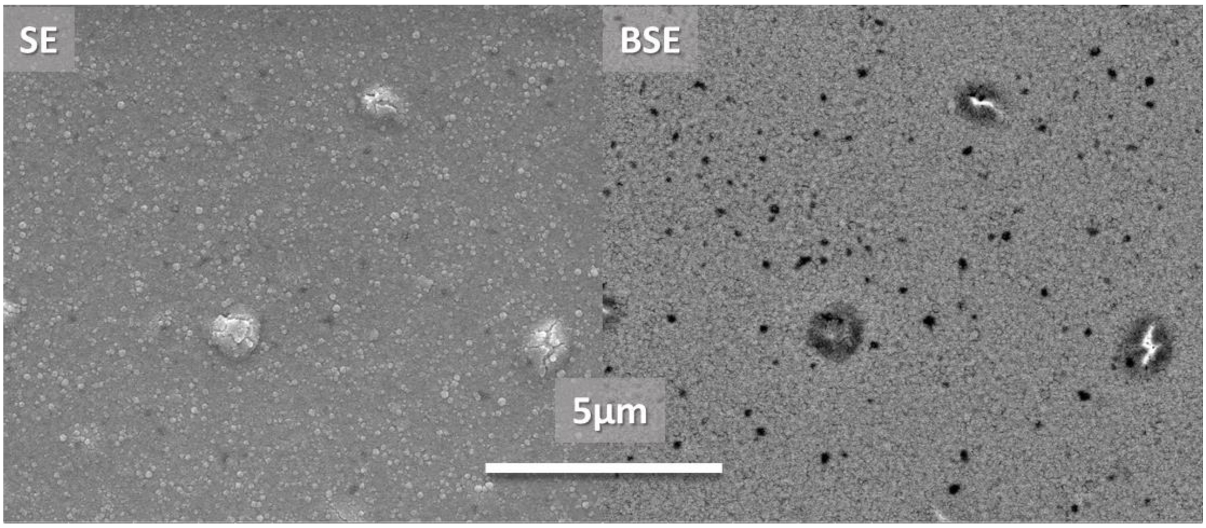

Figure S1: Surface images from secondary electrons (SE) and backscattered electrons (BSE) of a LMO film deposited by strategy I at 690 °C of maximum temperature, showing pores (black in BSE) and cracks (white in BSE) due to the dewetting of the Pt layer.

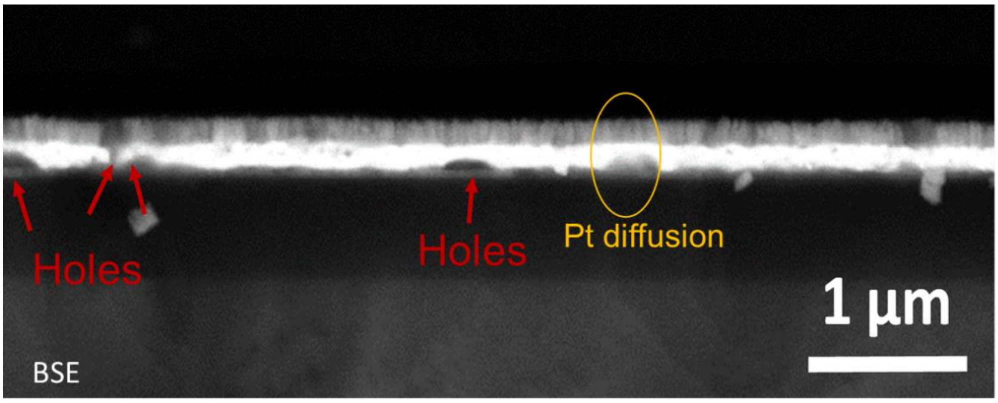

Figure S2: SEM cross section of the LMO thin film structure, deposited by strategy III (25000 pulses at 500 °C and 5000 pulses at 750 °C), showing the contrast in composition (BSE mode). The discontinuity in the Pt films is clearly visible forming holes and with Pt interdiffusion as indicated in the image. These holes provoke the appearance on pinholes at the LMO surface.

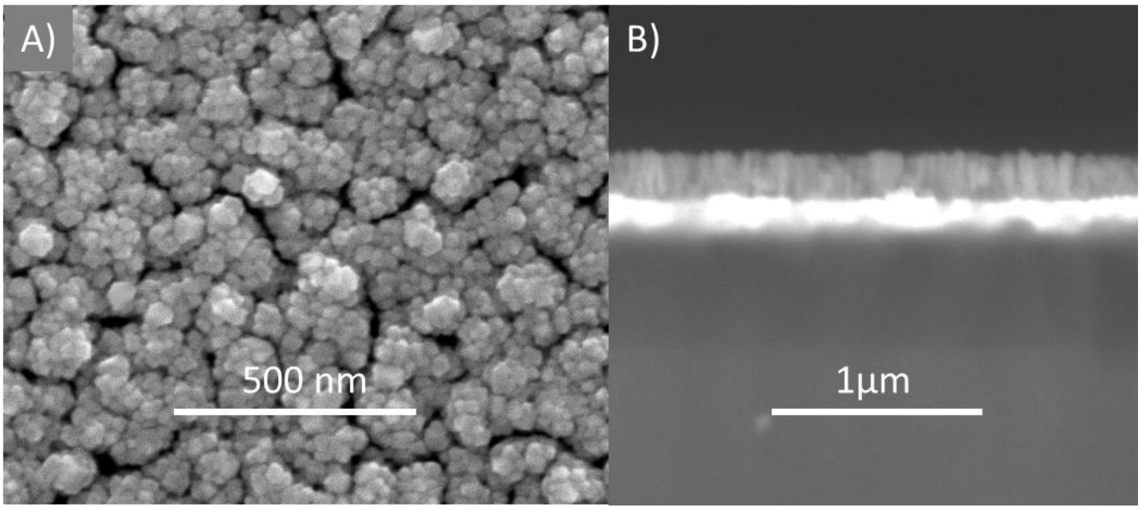

Figure S3: Columnar opening in a flower bouquet manner, in a sample deposited by strategy III (25000 pulses at 500 °C and 5000 pulses at 750 °C) A) SEM top view where the separation of the columns is visible. B) Columnar growth and opening cross section by BSE.
